# Schizont transcriptome variation among clinical isolates and laboratory-adapted clones of the malaria parasite *Plasmodium falciparum*

**DOI:** 10.1186/s12864-018-5257-x

**Published:** 2018-12-10

**Authors:** Sarah J. Tarr, Ofelia Díaz-Ingelmo, Lindsay B. Stewart, Suzanne E. Hocking, Lee Murray, Craig W. Duffy, Thomas D. Otto, Lia Chappell, Julian C. Rayner, Gordon A. Awandare, David J. Conway

**Affiliations:** 1 Pathogen Molecular Biology Department, London School of Hygiene and Tropical Medicine, London, UK; 2 Institute of Infection, Immunity and Inflammation, University of Glasgow, Scotland, UK; 3 Wellcome Sanger Institute, Hinxton, Cambridge, UK; 4 West African Centre for Cell Biology of Infectious Pathogens, Department of Biochemistry, Cell and Molecular Biology, University of Ghana, Legon, Ghana

**Keywords:** Eukaryotic microbial genomics, Biological replicates, Clinical samples, Culture adaptation, RNA-seq, Transcriptomic methods, Cell invasion, Immunity

## Abstract

**Background:**

Malaria parasites are genetically polymorphic and phenotypically plastic. In studying transcriptome variation among parasites from different infections, it is challenging to overcome potentially confounding technical and biological variation between samples. We investigate variation in the major human parasite *Plasmodium falciparum*, generating RNA-seq data on multiple independent replicate sample preparations of merozoite-containing intra-erythrocytic schizonts from a panel of clinical isolates and from long-term laboratory-adapted clones, with a goal of robustly identifying differentially expressed genes.

**Results:**

Analysis of biological sample replicates shows that increased numbers improve the true discovery rate of differentially expressed genes, and that six independent replicates of each parasite line allowed identification of most differences that could be detected with larger numbers. For highly expressed genes, focusing on the top quartile at schizont stages, there was more power to detect differences. Comparing cultured clinical isolates and laboratory-adapted clones, genes more highly expressed in the laboratory-adapted clones include those encoding an AP2 transcription factor (PF3D7_0420300), a ubiquitin-binding protein and two putative methyl transferases. In contrast, higher expression in clinical isolates was seen for the merozoite surface protein gene *dblmsp2*, proposed to be a marker of schizonts forming merozoites committed to sexual differentiation. Variable expression was extremely strongly, but not exclusively, associated with genes known to be targeted by Heterochromatin Protein 1. Clinical isolates show variable expression of several known merozoite invasion ligands, as well as other genes for which new RT-qPCR assays validate the quantitation and allow characterisation in samples with more limited material. Expression levels of these genes vary among schizont preparations of different clinical isolates in the first ex vivo cycle in patient erythrocytes, but mean levels are similar to those in continuously cultured clinical isolates.

**Conclusions:**

Analysis of multiple biological sample replicates greatly improves identification of genes variably expressed between different cultured parasite lines. Clinical isolates recently established in culture show differences from long-term adapted clones in transcript levels of particular genes, and are suitable for analyses requiring biological replicates to understand parasite phenotypes and variable expression likely to be relevant in nature.

**Electronic supplementary material:**

The online version of this article (10.1186/s12864-018-5257-x) contains supplementary material, which is available to authorized users.

## Introduction

Variation in gene expression is key to survival and reproduction of microbes, affecting diverse phenotypes such as sexual differentiation [1], adaptation to different growth conditions [2] and evasion of host immunity [3]. Malaria pathogenesis occurs as parasites undergo cycles of invasion and asexual replication inside erythrocytes. Towards the end of each cycle, schizonts develop which contain multiple merozoites that need to burst from the host cell and invade new erythrocytes. The parasite transcriptional program of the major human malaria parasite *Plasmodium falciparum* is highly synchronised throughout the asexual replication cycle in erythrocytes [4], but some variation exists between parasite clones [5, 6]. Analysis of naturally occurring polymorphism in *P. falciparum* has shown that selection maintains multiple alleles of many merozoite-stage genes [7, 8], some of which encode targets of naturally acquired immunity [9–11]. Genes expressed at this stage can also exhibit apparently pronounced variation in transcription among parasites [7, 12–14], but most analyses have been conducted on sample preparations that do not incorporate many biological replicates, so that the extent of variation among different parasite lines is unclear.

Accurate quantitation of differential gene expression between organisms or groups of organisms in whole-transcriptome analyses requires biological replicate sampling [15, 16]. The importance of minimising sampling error in order to detect real differential gene expression is widely recognised [17], and some bioinformatic tools can guide the determination of replicate numbers appropriate to experimental designs [18, 19]. In transcriptomic studies of malaria parasites, achieving large numbers of replicate sample preparations is difficult, particularly from clinical isolates [5], and most detailed understanding of transcription has been derived from a small number of parasite lines that have been grown in culture for many years. Although long-term culture-adapted parasites are phenotypically and transcriptionally diverse [6, 12, 20], it is not clear to what extent they reflect the diversity of parasites causing clinical malaria. Examination of genome sequences of culture-adapted parasites has identified premature stop codon mutants affecting some transcription factor genes and cell signalling protein genes [21, 22], as well as particular gene deletions and amplifications not seen in parasites sampled directly from patients [23–25].

Studies of parasite transcripts in clinical isolates face a challenge of interpreting the mixture of developmental stages that may be present in a blood sample, with parasites at different stages of the asexual cycle as well as parasites that have committed to sexual development [26–28]. Some of the unknown variation in parasite stage distribution may be accounted for using deconvolution methods in analysis [28, 29], and some random variation may be partly overcome statistically if it is possible to analyse large numbers of infections [30, 31]. However, *P. falciparum* parasites at the intra-erythrocytic schizont stage, containing merozoites ready to be released to invade other erythrocytes, are normally sequestered in organ capillaries and not present to be analysed in blood samples. This stage of the parasite may be studied by ex vivo development in the first cycle of following isolation from patients, and analysis of matured schizont stages has shown variable expression of particular genes [7, 13, 14], although the precision of such analyses are limited without biological replicate measurements.

Here we present gene expression profiles of schizont-stage malaria parasites from recently culture-established clinical isolates, and laboratory-adapted clones previously cultured for many years. We conduct RNA-seq analysis with multiple replicates of each parasite line, and show that high numbers of biological replicates improves the true-positive discovery rate for identifying differentially expressed genes. This identifies schizont-stage genes that have differing transcript levels between the long-term laboratory-adapted clones and cultured clinical isolates, as well as those variably expressed among the clinical isolates. The results confirm variable transcription in particular genes encoding ligands involved in erythrocyte invasion, and variation in expression of genes that have been less characterised was quantitatively validated by targeted RT-qPCR assays, allowing analysis to be extended to additional samples from which material is more limited. The expression levels of these genes in a panel of clinical isolates cultivated to schizont stage in the first ex vivo cycle were similar on average to those in continuously cultured clinical isolates. This encourages use of recently culture-established parasite lines for studies requiring biological replication.

## Results

### Biological replicate sampling improves detection of differentially expressed genes

RNA-seq paired-end short-read sequences from all samples were mapped to the *P. falciparum* strain 3D7 reference genome sequence, and regions of genes with high levels of allelic polymorphism were masked from analysis in order to minimize mapping bias among the different parasite lines (Additional file 1). Genes previously described as deleted in some parasites were not excluded, as most individual deletions are very rare [32] and exclusion would result in unnecessary loss of data for genes unaffected in the isolates sampled. After excluding the subtelomeric large gene families *var*, *rifin* and *stevor* for which short read sequence mapping is not generally locus-specific, a total of 5134 gene loci were analysed.

**Fig. 1:**
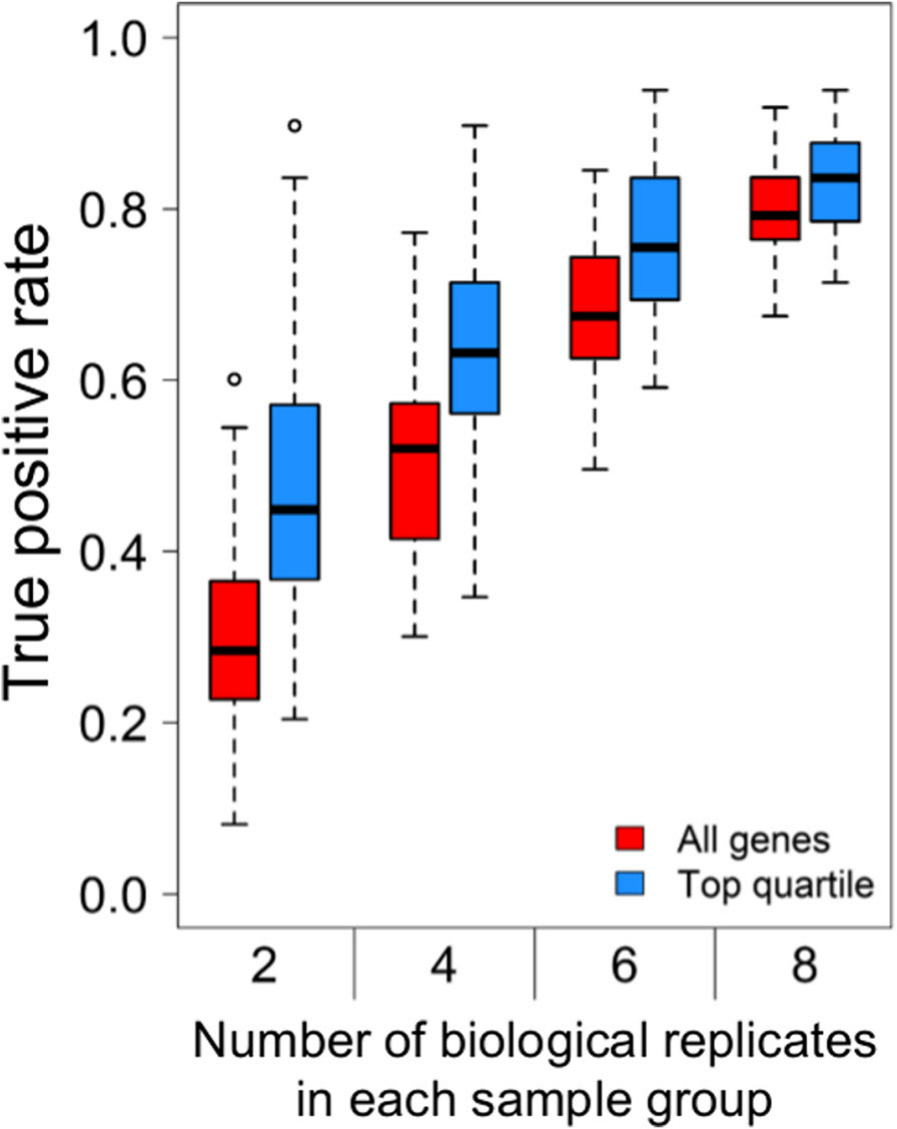
Increasing numbers of sample replicates improves identification of schizont-stage genes varying in expression between different *P. falciparum* lines. Assessment of the proportion of genes captured as being differentially expressed between two different parasite clones (3D7 and D10), by taking 100 random samples of two, four, six and eight replicates of each (out of ten initially analysed replicates that identified 123 genes with log_2_ differences of > 2 in relative transcript levels between the two clones)

We assessed the impact of numbers of biological replicates on detection of genes with differing transcript levels between parasite lines, by first analysing RNA-seq data for the 3D7 and D10 laboratory clones, focusing on schizonts purified by density gradient centrifugation from ten different culture preparations of each clone. Analysing data from all ten replicates identified 123 genes with adjusted *P* values < 0.01 and log_2_ differences of > 2 in relative transcript levels between the two clones (equivalent to at least 4-fold differences, Additional file 2). We assessed what proportions of these 123 genes were captured as being differentially expressed in 100 random samples of two, four, six and eight replicates within each group. The true-positive proportion of differentially expressed genes detected (using the same statistical criteria of absolute log_2_ fold difference > 2 and adjusted *P* value < 0.01) increased with the number of replicates within each group, median true-positive proportions being 0.28, 0.52, 0.67 and 0.79, for two, four, six and eight replicates respectively (Fig. 1). These true-positives accounted for the majority of genes indicated as differentially expressed, out of median totals of 42, 81, 108 and 111 genes, for two, four, six and eight replicates respectively. The median false-positive rates were very low in all cases (among the genes that did not show differences with 10 replicates, the proportions showing apparent differences with two, four, six and eight replicates were 0.001, 0.004, 0.005 and 0.004 respectively).

We also examined the sensitivity to detect differential expression when focusing specifically on genes that are highly expressed in schizont-stage samples, as these include genes most likely to be functionally important for the merozoite invasive stage. The FPKM values for each sample in each of the comparisons were averaged and the genes were ranked by their maximum expression level. The top quartile of most highly expressed genes were analysed for differences between clone 3D7 and D10, in all ten replicates and subsamples of lower replicate numbers, with 100 randomisations of each. In comparison with all ten replicates, there were median true-positive rates of 0.45, 0.63, 0.78 and 0.86 for comparisons respectively containing two, four, six and eight replicates, higher proportions than for the analysis of all genes (Fig. 1). Based on these data, it is evident that increasing biological replicate numbers for parasite RNA-seq improves discovery, and under our experimental conditions the use of six independent biological replicates detects the majority of genes shown to be differentially expressed using ten replicates.

### Comparison of gene expression in cultured clinical and laboratory-adapted isolates

**Fig. 2:**
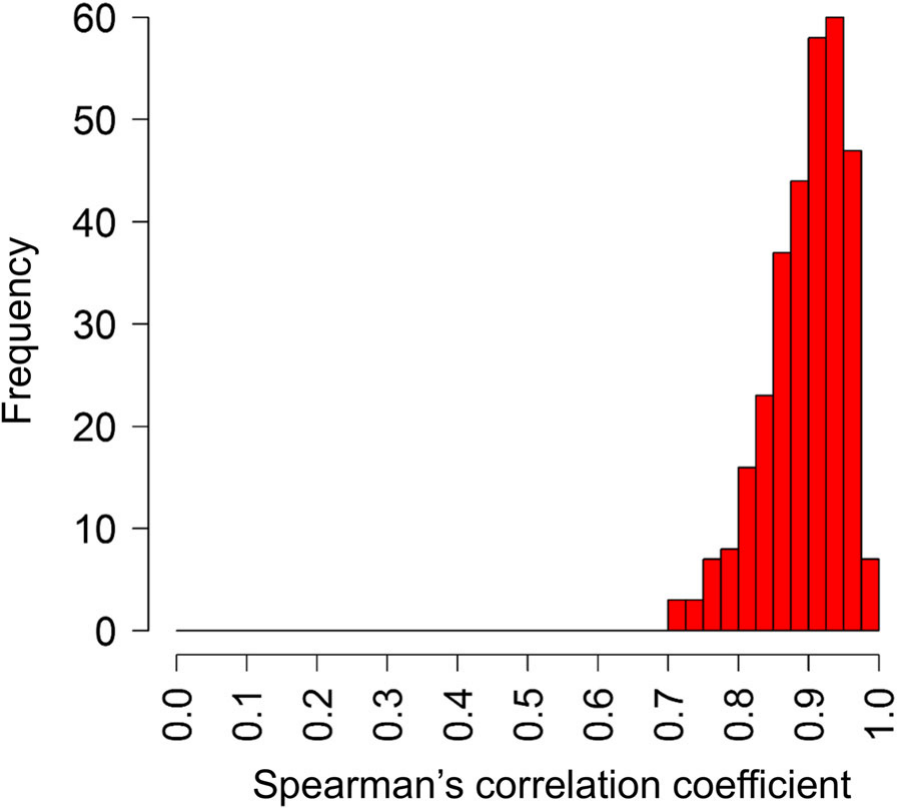
Distribution of correlations of FPKM expression values among biological sample replicate transcriptomes for each isolate or clone. The final numbers of replicates, and median Spearman’s correlation coefficients among replicates were: 3D7 *n* = 19, ρ = 0.89; D10 *n* = 10, ρ = 0.93; Dd2 *n* = 6, ρ = 0.95; HB3 *n* = 7, ρ = 0.89; 271 *n* = 3, ρ = 0.94; 278 *n* = 5, ρ = 0.91; 280 *n* = 6, ρ = 0.93; 286 *n* = 6, ρ = 0.97; 293 *n* = 6, ρ = 0.93; 296 *n* = 3, ρ = 0.90

In addition to the ten biological replicate preparations from each of clones 3D7 and D10 analysed above, another nine biological replicate preparations of schizonts from clone 3D7 were analysed, as well as seven biological replicates for each of two other laboratory-adapted clones (HB3 and Dd2), and five or six replicates of each of six cultured clinical isolates from Ghana (isolates 217, 278, 280, 286, 293 and 296: Additional file 3). Despite being enriched for mature schizont stages, multiple biological replicates cannot each have exactly the same distribution of parasite developmental stages, so to assess stage variability the RNA-seq read depth for each gene (FPKMs) for each replicate sample were correlated with FPKM values in reference RNA-seq data for seven cultured time points over a ~ 48 h *P. falciparum* cycle [33]. This identified only a small number of replicate samples that did not have a maximum Spearman’s correlation with parasites at schizont stages (either 40 or 48 h post-invasion), and these were excluded from further analyses (Additional file 3). Although there is variation evident among the remaining replicates that were analysed, within each isolate the FPKMs among replicates correlated highly, with average Spearman’s ρ > 0.95 for pairwise correlations across all genes (Fig. 2).

**Fig. 3:**
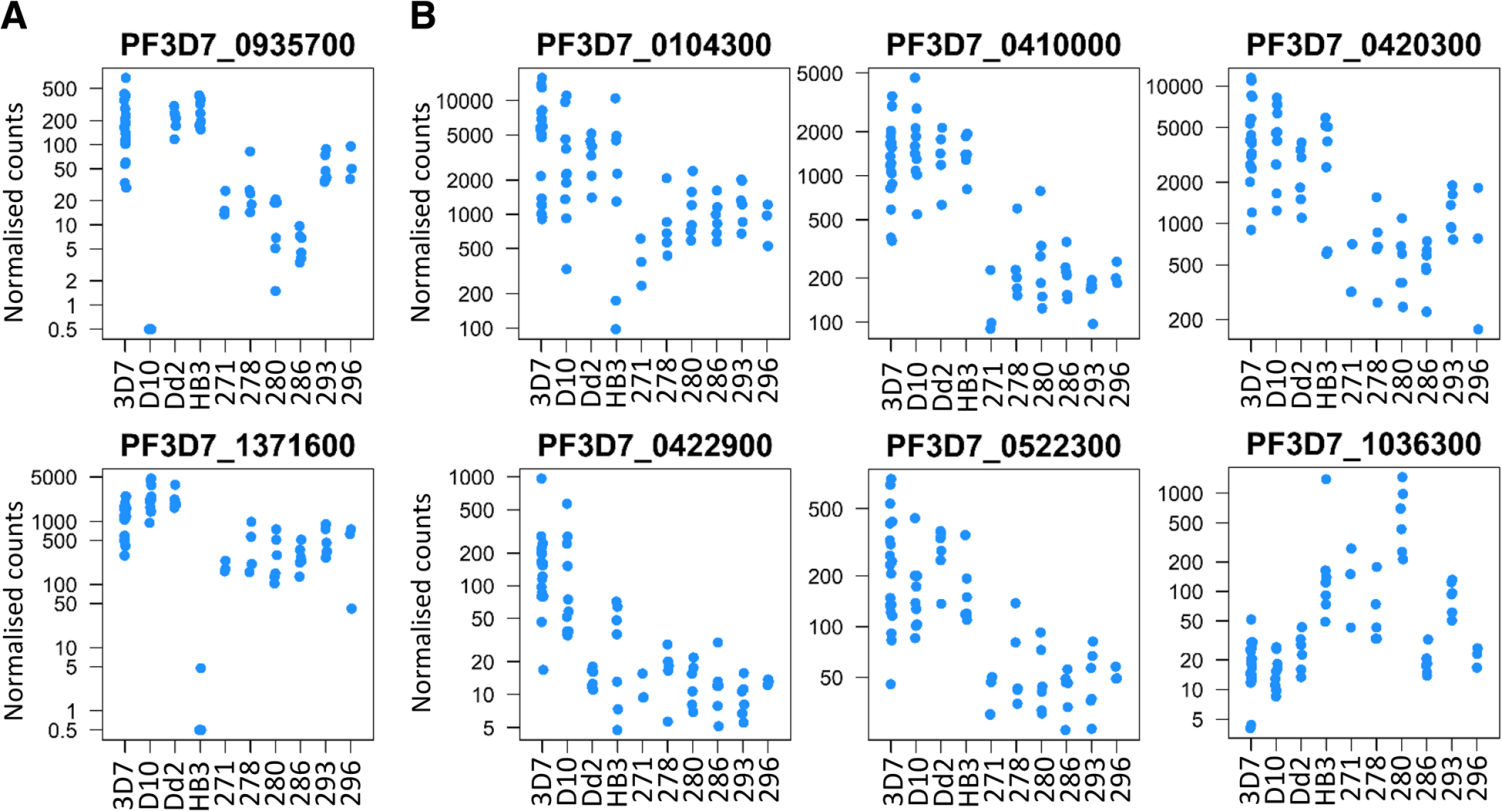
Gene transcript levels among preparations of schizont-enriched *P. falciparum* cultures of laboratory-adapted clones and clinical isolates. Plots of normalised RNA-seq read counts for eight of the genes showing differences between four long-term laboratory-adapted clones (3D7, D10, Dd2 and HB3) and six recently culture-established Ghanaian clinical isolates (271, 278, 280, 286, 293 and 296). Each point shows the value for an independent biological replicate sample. For all genes, a wide variation in transcript levels among biological replicates may be noted. **a** The two panels show examples of genes for which one of the laboratory-adapted clones had a deletion, D10 which is missing the PF3D7_0935700 gene as part of a deleted region near the end of chromosome 9, and HB3 which is missing gene PF3D7_1371600 that encodes erythrocyte binding-like protein 1. These, and other genes with suspected or known deletions in the sampled parasites, are excluded from the list of differentially expressed genes in Table 1 but are listed Additional file 4: Table S2. **b** The six panels show examples of genes listed in Table 1 that are in the top quartile of expression and show a log_2_ fold difference > 2 between the groups. Most of these genes have lower transcript levels in the clinical isolates (including AP2 transcription factor gene PF3D7_0420300, as well as predicted methyltransferase genes PF3D7_0422900 and PF3D7_0522300, and ubiquitin-binding protein 1 gene PF3D7_0104300). In contrast, the *mspdbl2* gene PF3D7_1036300 shown in the bottom right panel had higher transcript levels in clinical isolates

In an overall comparison of the schizont preparations from four long-term laboratory-adapted clones and six cultured clinical isolates, 132 genes (2.6% of those analysed) appeared to have different transcript levels between the two groups, each having log_2_ fold difference > 2 and adjusted *P* value < 0.01 (Additional file 4). Among the genes within the top quartile of expression values overall, 18 genes (1.4%) had such a difference between the groups, a lower proportion than among the rest of the genes (Odds Ratio 0.52, Fisher’s exact *P* = 0.006). Examining the 18 genes with differing transcript levels among the top quartile of expressed genes, several were seen to contain a deletion in one of the parasite lines or to be members of multigene families. For example, PF3D7_1371600 (*ebl-1*) and PF3D7_0935700 (near the end of chromosome 9) are deleted in the HB3 strain and D10 strain respectively (Fig. 3), as previously documented [34, 35]. After excluding these genes and others for which one or more of the samples were presumed to contain genetic deletions, and excluding members of sub-telomeric multi-gene families, ten highly expressed single-locus genes showed differences between laboratory and clinical isolates (Table 1).

**Table 1:** *P. falciparum* genes in the top quartile of expression showing a difference in schizont stage transcript levels between long-term laboratory-adapted clones and recent clinical isolates in culture

Gene ID	Product annotation	Log_2_ fold difference	Wald test
Higher transcription in long-term laboratory-adapted clones			
PF3D7_0422900	methyltransferase, putative	−2.50	−6.48
PF3D7_0501300	skeleton-binding protein 1	−2.50	−4.98
PF3D7_0410000	erythrocyte vesicle protein 1	−2.38	−10.62
PF3D7_0104300	ubiquitin carboxyl-terminal hydrolase 1, putative	−2.18	−6.13
PF3D7_0522300	18S rRNA (guanine-N(7))-methyltransferase, putative	−2.15	−8.67
PF3D7_0420300	AP2 domain transcription factor, putative	−2.02	−7.19
PF3D7_1030200	claudin-like apicomplexan microneme protein, putative	−2.00	−9.21
PF3D7_1327300	conserved *Plasmodium* protein, unknown function	−2.00	−4.97
Higher transcription in clinical isolates			
PF3D7_1036300	duffy binding-like merozoite surface protein 2	3.02	5.32
PF3D7_1302100	gamete antigen 27/25	2.02	3.99

There was considerable variation in the measurements for individual genes among the different biological replicate preparations of each parasite clone or isolate, with five to ten-fold ranges of levels being not unusual (Fig. 3), illustrating the importance of sampling and analysing multiple biological replicates. To check for potential effects of fine differences in schizont maturity among samples, expression levels of the ten most differentiated genes were compared between samples having overall highest correlations with either the 40-h or the 48-h time points of a reference transcriptome dataset [33]. This showed that the gene expression distributions were not due to differences in estimated maturity, and that differences between clinical isolates and laboratory clones remained when only analysing replicates having peak correlation with the 40-h time point (Additional file 5).

Eight of the ten highly expressed genes showing a difference between the groups had higher transcript levels in the long-term laboratory-adapted clones than in the cultured clinical isolates (Table 1). These include an AP2 transcription factor gene PF3D7_0420300, as well as two predicted methyltransferase genes (PF3D7_0422900 and PF3D7_0522300), and gene PF3D7_0104300 encoding ubiquitin-binding protein 1 which is involved in protein turnover [36] (Fig. 3). The genes that had higher transcript levels in clinical isolates were PF3D7_1036300 which encodes the merozoite surface protein MSPDBL2 (Fig. 3), and PF3D7_1302100 which encodes the gamete antigen 27/25. MSPDBL2 has previously been shown to be only expressed in a minority of schizonts in culture, with clone HB3 having more positive schizonts than other laboratory-adapted clones [7], consistent with the higher transcript level in HB3 seen here and previously. Recent experimental analysis has shown *mspdbl2* transcript levels to be markedly increased in parasite cultures when function of heterochromatin protein 1 (HP1) is suppressed by gametocyte development protein 1 (GDV1) leading to gametocytogenesis [37, 38]. Although gamete antigen 27/25 had higher transcript levels in clinical isolates and was previously shown to be expressed in early gametocytes [38, 39] another gene PF3D7_1327300 that is transcribed in gametocytes [40] showed lower expression in the clinical isolates (Table 1).

We investigated whether there was an overall association between the genes differentially expressed and targets of HP1 regulation [37, 41]. Of the HP1-regulated genes previously identified [37], 118 genes are among those analysed here (*var*, *rifin* and *stevor* gene families were excluded). Of the 132 genes differentially expressed between the two groups, 24 (18.2%) were targets of HP1 regulation, a higher proportion than among the rest of the genes (Odds Ratio 11.3, Fisher’s exact *P* = 1.3 × 10^− 5^).

### Differential expression of schizont-stage genes among cultured clinical isolates

Overall, 214 genes (4.2%) showed a log_2_ fold difference > 2 for at least one of the 15 pairwise comparisons among cultured clinical isolates. Of those genes in the top quartile of expression levels, 39 genes (3.0%) showed a difference for at least one pairwise comparison, a lower proportion than among the less expressed genes (Odds Ratio 0.66, Fisher’s Exact *P* = 0.02) (Additional file 6). Some of the variably expressed genes encode merozoite proteins previously characterised as transcriptionally variable among ex vivo clinical isolates [14], including *dblmsp2* and *msp6*, as well as erythrocyte binding antigen genes *eba-175*, *eba-181*, and *eba-140* (which is also deleted in one of the laboratory adapted clones) (Additional file 7). Notably, among the 214 genes variably expressed among cultured clinical isolates, 38 (17.8%) have been shown to be targets of HP1 regulation, compared with only 1.6% of the non-variably expressed genes (Odds Ratio 12.9, Fisher’s Exact *P* < 2.2 × 10^− 16^). This association was even more extreme among genes in the top quartile of overall expression values, 16 (41%) of the 39 variably expressed genes being targets of HP1 regulation compared with only 0.6% of the non-variably expressed genes (Odds Ratio 118.6, Fisher’s exact *P* < 2.2 × 10^− 16^).

**Fig. 4:**
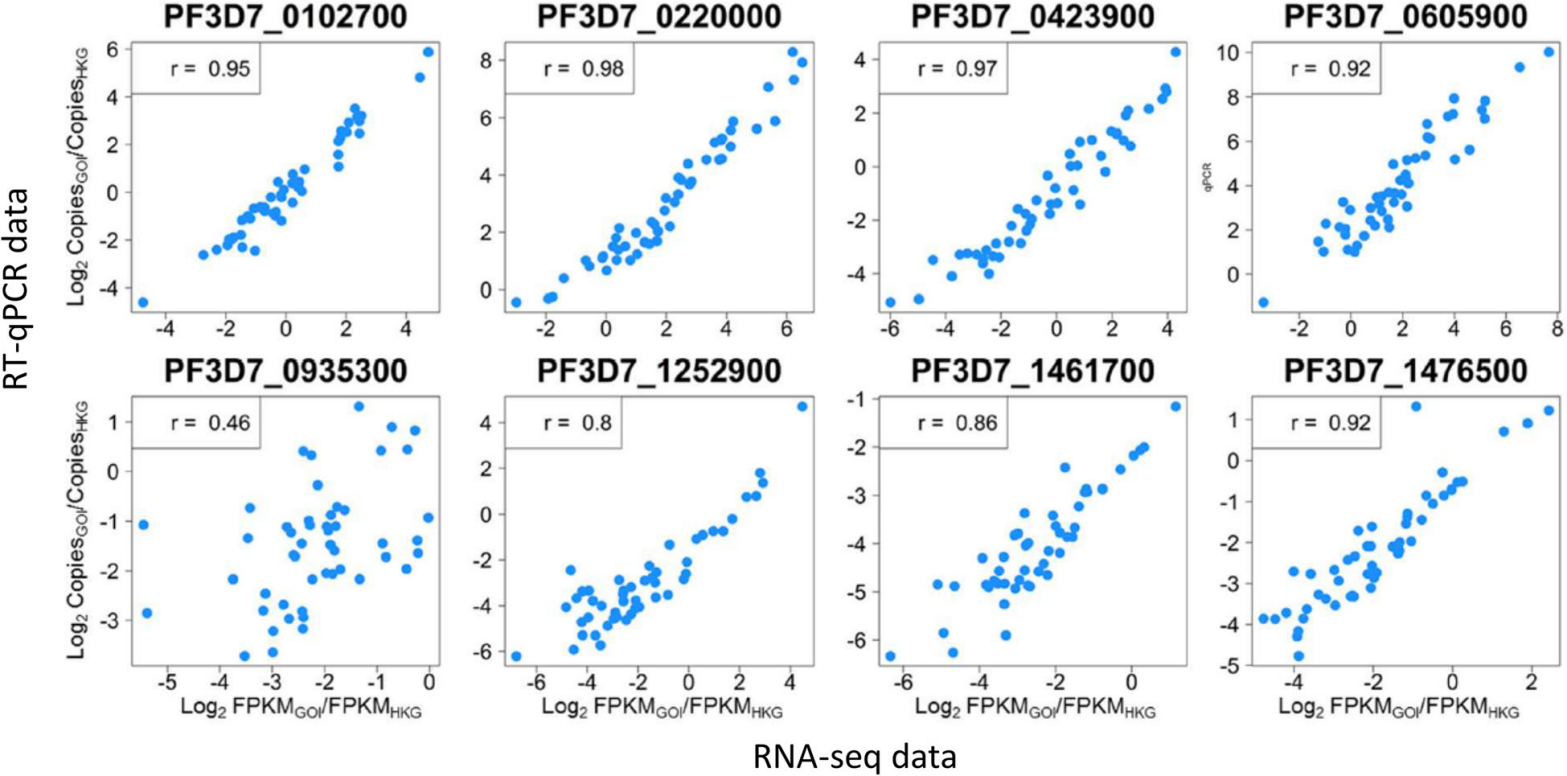
High correlations between RNA-seq and RT-qPCR expression measures. Eight genes identified as differentially expressed among clinical isolates by RNA-seq were validated by RT-qPCR for 49 of the independent RNA preparations from the four laboratory-adapted clones and six cultured clinical isolates under study. Scatter plots for each gene show the RT-qPCR gene of interest (GOI) copies (log_2_-transformed) normalised to house-keeping gene copies (HKG; PF3D7_0717700), correlated with the RNA-seq GOI FPKM values (log_2_-transformed) normalised to HKG FPKM values. Spearman’s correlation coefficients are shown for each plot

To validate the data obtained through RNA-seq, a subset of genes was selected for quantitation by reverse-transcription quantitative PCR (RT-qPCR). We chose differentially expressed genes encoding proteins containing predicted transmembrane domains or signal peptides, thereby likely to enter the parasite secretory pathway, excluding genes previously characterised as variably expressed or members of multi-gene families. Eight genes were selected for assay (Additional file 8), two of which encode known antigens (merozoite-associated tryptophan-rich antigen [42] and liver-stage antigen-3 [43]). These gene transcripts were quantified by RT-qPCR in 49 RNA preparations from the laboratory clones and clinical isolates that had been analysed by RNA-seq. Transcript levels were normalised to those of a housekeeping gene (serine-tRNA ligase PF3D7_0717700) and correlated with similarly normalised FPKM expression values for the RNA-seq data. Each gene showed strong positive and highly significant correlation between RNA-seq and RT-qPCR-derived expression measures, all except one having correlation coefficients > 0.8 (Fig. 4).

### Variable expression among isolates with schizonts sampled in the first ex vivo cycle

**Fig. 5:**
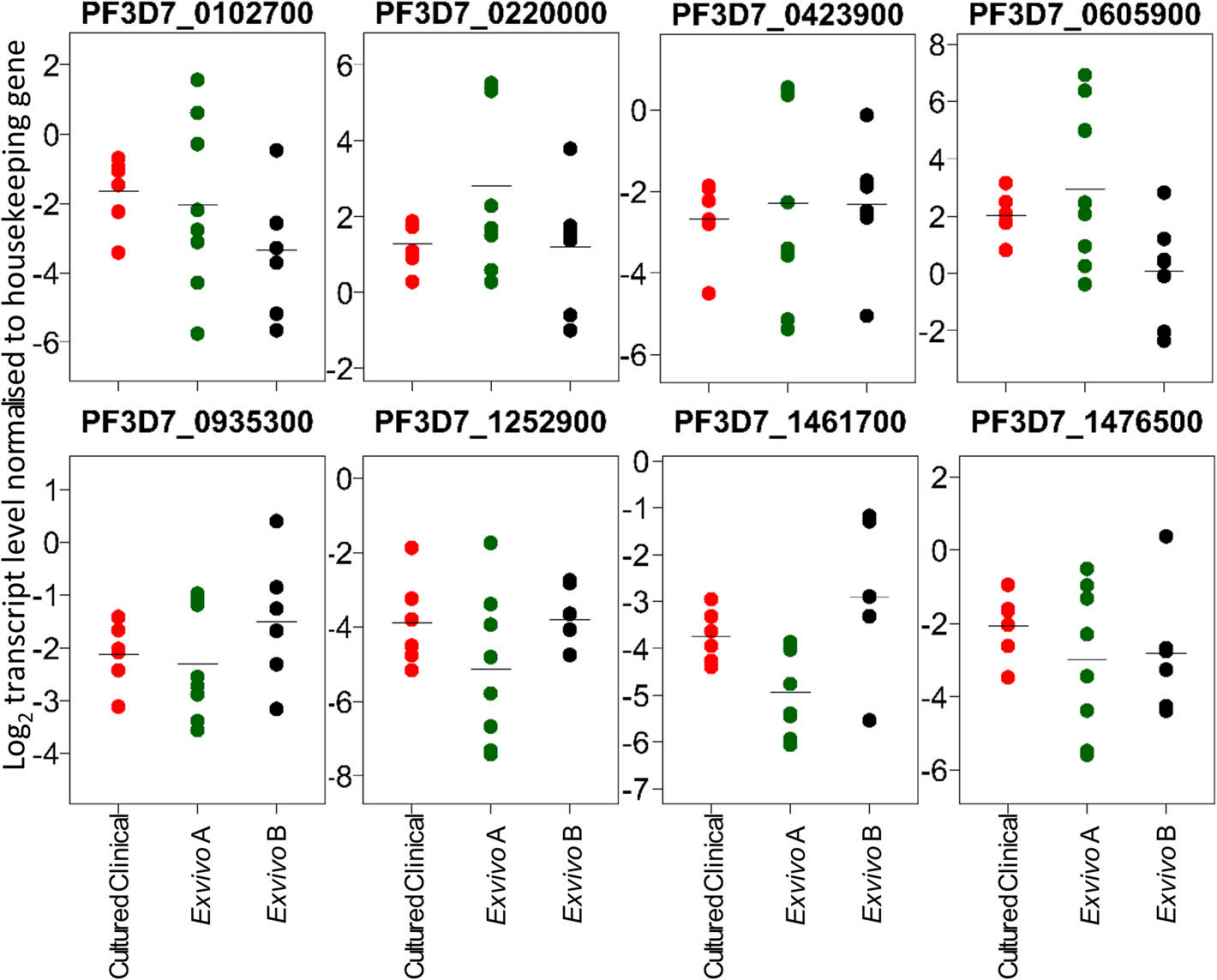
Transcript levels of variably expressed genes for first cycle ex vivo clinical samples compared with continuously cultured isolates. For each gene, the log_2_ normalised expression value is plotted as a ratio of the value for a housekeeping gene (PF3D7_0717700). For the cultured clinical isolates (red, *N* = 6), each point represents the mean expression value for each gene calculated across multiple replicates of each isolate. For the ex vivo clinical isolates, each point represents values for single samples without replicates. The panel of ex vivo A samples (green *N* = 8) were analysed by RT-qPCR, while the panel of ex vivo B samples (black, *N* = 7) were analysed by RNA-seq

We next determined the expression levels in additional clinical samples from Ghana matured during the first cycle ex vivo until a large proportion of parasites were schizonts. For nine of these isolates there was sufficient RNA yield for RNA-seq to be performed, out of which seven gave adequate data for transcriptome analysis. With remaining material from two of these as well as another six isolates there was sufficient to analyse the selected eight gene panel by RT-qPCR. Characterisation of the ex vivo samples showed considerable variation in relative expression levels of each gene, but the mean levels across the isolates were similar to those determined in the continuously-cultured parasites that had extensive sample replication (Fig. 5). Without sample replication of the first cycle ex vivo clinical samples, routine technical variation and random sampling effects are likely to exaggerate the apparent variation in expression levels. However, the observation that for each gene the normalised expression values have similar means to those for cultured clinical lines indicates there are no systematic differences in expression between the first ex vivo cycle and under continuous culture conditions. These results support the use of cultured clinical isolates as a means of studying parasite gene expression reflecting original populations, while enabling the necessary experimental replication for many analyses.

## Discussion

Transcriptomic analyses perform optimally with increased levels of sample replication to minimise the impact of stochastic or technical variation in individual samples [17, 44]. This is particularly important for studies focusing on a defined developmental stage, as errors may be caused by subtle differences between samples. However, the nature of clinical samples of malaria parasites is such that replication is technically very difficult. In order to assess the degree of replication useful to identify differentially expressed genes, we first generated RNA-seq profiles from multiple replicates of laboratory-adapted clones, and used the data to calculate the true- and false-discovery rates obtained using fewer replicates. Consistent with studies on other cultured eukaryotes [17], we show that for malaria parasites increasing the sample replication improves the true-positive discovery rate in identifying variably expressed genes, and under the experimental conditions here six independent replicates were useful to balance accuracy and experimental feasibility. Where these numbers of replicates are not obtainable, it is recommended to focus on genes that are highly expressed in order to achieve as much accuracy as possible per gene. Conversely, where a very high sensitivity is needed it may be worth studying even more biological replicates for each parasite line and condition being investigated.

It has been proposed that spontaneous transcriptional variation within parasite populations is a strategy that ensures fitness of parasites facing a range of potential and changing selective pressures [6], and it is likely that the rates and mechanisms of regulation differ among most genes. In comparison between long-term laboratory-adapted clones and cultured clinical isolates, we found that among the genes most differentially expressed were an AP2-transcription factor [45] and two methyltransferases, the functional roles of which remain to be determined although genome-wide mutagenesis studies indicate they are important for *P. falciparum* asexual erythrocytic stage replication [46]. It is possible that use of different oxygen concentrations in the culture of long-term adapted clones and recent clinical isolates here might affect transcription of some genes, particularly in earlier trophic stages of the intra-erythrocytic cycle [47], and although we did not consider oxygen metabolism in analysis of schizont stages it could be examined in future. Most of the differentially expressed genes noted here had lower transcription in the clinical isolates, and we note that HP1 is responsible for extensive gene silencing in malaria parasites [38], and there was an over representation of HP1-regulated genes among those differentially expressed between laboratory-adapted clones and cultured clinical isolates. It would be interesting to undertake studies directly on HP1 and other chromatin modifications in recent clinical isolates, as experimental studies on chromatin have so far focused on long-term laboratory-adapted clones. Interestingly, among the top quartile of expressed genes, the one most markedly highly expressed in clinical isolates was *mspdbl2* that encodes a merozoite surface protein expressed within only a minority of schizonts [7] which may be a marker of parasites committed to sexual development [37]. Further studies of clinical isolates are needed to understand the process of parasite commitment to sexual differentiation and transmission, as indicated by other transcriptome comparisons [5, 30].

We identified many genes differentially expressed among the clinical isolates. Targeted RT-qPCR assays of schizont-stage ex vivo cultured clinical isolates have previously shown some of the genes to be differentially expressed [7, 13, 14]. Focusing on genes for which variable expression has not been previously studied, we selected a panel of eight highly differentially expressed genes that encode secreted proteins. Of these, the merozoite-associated tryptophan-rich antigen and liver stage antigen 3 have been previously identified as merozoite proteins [42, 43], and another gene (PF3D7_0423900) is adjacent to loci encoding the cysteine-rich protective antigen (CyRPA) and reticulocyte binding homologue 5 (Rh5) which are targets of antibodies that inhibit merozoite invasion. We extensively validated the RNA-seq data by RT-qPCR for these genes, and extended quantitation to additional ex vivo clinical isolate samples. Despite the lack of sample replicates for the latter, results showed that average expression levels for each gene were similar to those for the cultured samples that had biological replicates.

Together, these data highlight new candidates for investigation as potential determinants of alternative developmental pathways or targets of immunity. Characterisation of gene transcript levels is only one level of analysis, as proteomic characterisation [48] and analysis of phosphorylation and other protein modifications [49, 50] may also identify functional determinants, and large-scale analysis of parasite protein variation has not yet been attempted on clinical isolates. A more complete description of transcriptome variation may also be derived in future by analysis of individual infected cells, which has not been undertaken on parasites from clinical isolates, as initial single-cell sequencing studies of laboratory model malaria parasites have only recently begun to probe the expression pathways of different life-cycle stages [51–53]. Scaling up transcriptomic analyses to clinical studies is challenging, and biological replicate preparations of parasite samples may only be feasible in a limited number of cases. In an epidemiological context, reduction of error also requires large sample sizes and tight definition of clinical phenotypes, and more statistical analysis and modelling of covariates may be appropriate, ideally including data on host transcriptome variation within the same samples [54].

## Conclusions

The transcriptomes of *Plasmodium falciparum* erythrocytic schizont preparations of long-term laboratory-adapted clones and recently established clinical isolates were analysed, using large numbers of biological replicate samples to minimise the impact of inter-replicate variation on observed patterns of differential expression. Testing subsamples of up to ten replicates of each cultured line shows that six replicate preparations gives power to detect most differences. Particular genes show differential expression in laboratory-adapted clones compared to cultured clinical isolates, or among different clinical isolates, including those predicted to affect differentiation and encoding targets of immunity. In schizonts grown in the first cycle ex vivo, expression levels of a panel of these genes vary among isolates, but mean levels are similar to those in continuously cultured clinical isolates suitable for experimental analyses requiring biological replicates to understand relevant parasite phenotypes.

## Methods

### *P. falciparum* isolates sampled from clinical malaria cases

Blood samples were collected from clinical malaria cases attending Ghana government health facilities between 2012 and 2013, in Kintampo (Brong-Ahafo Region of central Ghana), and Navrongo (Kassena-Nankana East Municipality, in the Upper East Region of northern Ghana). Patients were eligible to participate in the study if they had uncomplicated clinical malaria, were aged 2–14 years, tested positive for *P. falciparum* malaria by lateral flow rapid diagnostic test and slide microscopy, and had not taken antimalarial drugs during the 72 h preceding sample collection. Venous blood samples (up to 5 ml) were collected into heparinized Vacutainer tubes (BD Biosciences). Blood samples were centrifuged, plasma separated and leukocyte buffy coat cell layer removed, and erythrocytes were cryopreserved in glycerolyte and stored frozen at − 80 °C or in liquid nitrogen until shipment on dry ice to the London School of Hygiene and Tropical Medicine for subsequent culture.

### Parasite culture and enrichment for schizont stages

Parasites from ex vivo clinical samples were matured within the original patient erythrocytes at 2–5% haematoctit for up to 48 h in RPMI 1640 medium containing 2% human AB serum (GE Healthcare) and 0.3% Albumax II (Thermo Fisher Scientific) under an atmosphere of 5% O_2_, 5% CO_2_, and 90% N_2_ at 37 °C, until most parasites were at the schizont stage of parasite development. Laboratory-adapted clones and continuously cultured clinical isolates were grown in erythrocytes at 2–5% haematocrit in RPMI 1640 medium containing 0.5% Albumax II, at 37 °C. The biological replicate schizont preparations of clinical isolates for RNA-seq analysis were made by sampling at multiple time points between 182 and 250 days after first initiation into continuous culture. Laboratory-adapted clones which have been in culture for many years (3D7, HB3, Dd2, and D10) were maintained under atmospheric air with 5% CO_2_, and cultured clinical isolates were maintained in 5% O_2_, 5% CO_2_, 90% N_2_. Schizonts were purified using magnetic-activated cell sorting (MACS) for all of the lines, except for the D10 and HB3 clones and ten of the 3D7 replicates which were purified using Percoll density gradient centrifugation.

For MACS purification of cultured clinical isolates, parasites were prepared from 100 ml cultures with at least 0.7% of erythrocytes containing schizonts. Parasites were isolated by magnetic purification using magnetic LD Separation columns (Miltenyi Biotech). One column was used per 25 ml culture, and first washed twice in 3 ml of culture medium at room temperature. Parasite culture erythrocytes were pelleted by centrifugation at 500 g for 5 min, then re-suspended in 3 ml culture medium per 1 ml of packed cell volume. The re-suspended material was bound to the MACS column, which was then washed three times with 3 ml culture medium, and schizonts were eluted twice by removing the magnet from the column and forcing 2 ml culture medium through the column into a 15 ml collection tube. Finally, the schizonts were pelleted by centrifugation at 500 g for 5 min and the pellet volume was estimated, with 0.5 μl used for Giemsa-stained microscopical examination to assess staging, 1 μl added back to 250 μl culture at 0.8% hematocrit to follow the progression. Remaining parasites were re-suspended in 1.5 ml of culture medium with 10 μM E64 in a 12 well plate, and parasites were incubated for 5.5 h in 5% CO_2_ at 37 °C, before centrifugation in a 1.5 ml tube and processing for RNA extraction.

For discontinuous density centrifugation purification, parasites were maintained as 25 ml cultures at 2.5% hematocrit. Cultures were used when > 1% erythrocytes contained parasites with multiple nuclei. Schizonts were purified using 70% Percoll (GE Healthcare)/2.93% sorbitol/PBS overlayed with 35% Percoll/1.47% sorbitol/PBS, which was overlayed with parasitized erythrocytes, allowing the schizonts to be separated by centrifugation at 2500 g for 10 min at 24 °C, with a light brake at the end of centrifugation. Purified schizonts were washed once in complete medium and the pellet volume was estimated, following which the pellet was resuspended with six times the pellet volume of 50% haematocrit erythrocytes, a slide was prepared for microscopic examination, and the cells were then incubated in 6 ml of complete culture medium. Of this, 1 ml was used as a control untreated sample to track parasite egress, and 10 μM final concentration of E64 was added to the remaining 5 ml, and both were cultured at 37 °C in a 5% CO_2_ static incubator for 5.5 h. Schizonts from the E64-treated culture were overlaid on 70% Percoll/2.93% sorbitol/PBS and separated by centrifugation at 2500 g for 10 min at 24 °C, with a light brake at the end of centrifugation. The schizont layer was washed once in complete culture medium and final cell pellets of approximately 10–20 μl were used for RNA extraction.

### RNA extraction

Erythrocytes containing the prepared schizonts were resuspended in 500 μl TRIzol® reagent (Thermo Fisher Scientific) and stored at − 80 °C until RNA extraction using RNeasy mini columns (Qiagen). RNA pellets were dissolved in 100 μl RNase-free H_2_O, and a second RNA clean-up and on-column DNase treatment was carried out with RNA eluted in 30–50 μl RNase-free H_2_O, and concentration quantified by Qubit High Sensitivity RNA Assay (Thermo Fisher Scientific). Samples containing at least 500 ng RNA were considered for RNA-seq processing after the RNA integrity was checked on an Agilent Bioanalyzer using RNA 6000 Nano reagents and chips (Agilent Genomics).

### RNA-seq library preparation and sequencing

RNA-seq libraries of each of the replicate preparations of parasites were prepared with TruSeq Stranded mRNA Library Prep Kits (Illumina) using 500 ng – 1 μg RNA following the Illumina TruSeq Stranded mRNA protocol. Libraries were validated on an Agilent Bioanalyzer using DNA 1000 reagents and chips (Agilent Genomics) to quantify library sizes and confirm the absence of primer dimers. Libraries were quantified using a KAPA Universal Library Quantification kit (Roche Diagnostics Limited) on a 7500 Fast Real-Time PCR System (Thermo Fisher Scientific) and library concentrations were adjusted for library size. 12–15 pM pooled libraries were sequenced on a MiSeq System (Illumina) using a MiSeq Reagent Kit v3 (Illumina) with 2 × 75 cycles.

RNA-seq libraries of ex vivo schizont-enriched *P. falciparum* isolates (not cultured beyond the first cycle) were prepared using a modified protocol. PolyA+ RNA (mRNA) was selected using magnetic oligo-d(T) beads, and mRNA was reverse transcribed using Superscript III® (Thermo Fisher Scientific), primed using oligo-d(T) primers, with dUTP included during second-strand cDNA synthesis. The resulting double stranded cDNA was fragmented using a Covaris AFA sonicator. Sheared double stranded cDNA was dA-tailed, end repaired, and “PCR-free” barcoded sequencing adaptors (Bioo Scientific) [55] were ligated. Libraries were cleaned twice, using solid phase reversible immobilisation beads, and eluted in EB buffer (Qiagen). Second strand cDNA was removed using uracil-specific excision reagent enzyme mix (NEB) prior to library generation, and libraries were assayed by quantitative PCR prior to sequencing on a HiSeq system (Illumina).

### Data analysis

Raw Illumina sequence reads were aligned to the *P. falciparum* 3D7 v3 genome using HISAT2 [56] and converted to ‘BAM’ format using SAMtools [57]. Reads with MAPQ scores < 60 were removed. Reads were counted using the “summarizeOverlaps” feature of the GenomicAlignments package [58] in R, against a previously published *P. falciparum* genome annotation file that had been masked for regions of polymorphism (outlined in Additional file 1, which explains the coded annotation in Additional files 9 and 10). Fragments Per Kilobase of transcript per Million mapped reads (FPKMs) for the data here and those of previously published data on a developmental time course of the clone 3D7 [33] were calculated using the ‘fpkm’ function of DESeq2 [59] in R. FPKMs for all genes in each of our parasite preparations were correlated using a Spearman’s Rank correlation with FPKMs for each of the seven time points (0, 8, 16, 24, 32, 40 and 48 h post-invasion; Additional file 2: Table S1). Replicates with peak Spearman correlation values of ρ > 0.7 at the latest time points (40 or 48 h) were included for further analysis. Differential expression analysis was conducted using DESeq2 in R.

To test that the protocol for E64 treatment to prevent schizont egress from erythrocytes had no major effect on the transcriptomes, a comparison of four paired replicates of schizont preparations of cultured *P. falciparum* clone 3D7 was performed, which showed that only a single gene had log_2_ fold difference > 2 (Additional file 11), a difference considered as potentially random and marginal compared to differences anticipated between different parasite lines. The E64 treatment as described above enabled efficient preparation of mature schizonts, so this was used for preparations of schizonts throughout the study.

### RT-qPCR assays

150–500 ng total RNA from each preparation of parasite schizonts was reverse transcribed using Superscript II® (Thermo Fisher Scientific) with 250 ng random hexamer oligonucleotide primers per 20 μl reaction. Quantitative PCR (qPCR) was carried out using SYBR® Select Master Mix (Thermo Fisher Scientific) with 500 nM forward and reverse primers, in a Prism 7500 Fast qPCR machine. For each gene, threshold-cycle values were quantified against a serially diluted genomic DNA (Dd2 strain) standard curve, run on the same plate. Cycling parameters were: 50 °C for 2 min, 95 °C for 2 min followed by 40 cycles of 95 °C for 15 s and 60 °C for 1 min. All wells were run as 10 μl volumes in technical duplicate. The qPCR copy numbers were normalised against copies of a house-keeping gene, PF3D7_0717700 [60]. PCR primer pair sequences are as follows: PF3D7_0102700 5’-CAACCAGACAAACAACAAGAAA-3′ and 5’-TCCATTCTGATGCTTTCCAC-3′, PF3D7_0220000 5’-GTAAATGGTGAATTAGCTAGTGAAG-3′ and 5’-CCTTTATATCTTCGGCTTCTTCT-3′, PF3D7_0423900 5’-GAGAAGAAGCCAAAGTAAATGAAC-3′ and 5’-GAATGTGTCAAGTGCATCATAA-3′, PF3D7_0605900 5’-CGCAATAACAAGAAGTCACAAA-3′ and 5′-AAGATTGTAGGAGGGTGTTAAT-3′, PF3D7_0935300 5’-GGGCTGCTGTTATACCTTG-3′ and 5’-AGAATGAGGAGGTTCAGTTTG-3′, PF3D7_1252900 5’-CCTTAGTAGAACTTCAAAGTGACA-3′ and 5’-TGTAACCAACTACGAAATTCCC-3′, PF3D7_1461700 5’-TGCTGACTTTCAAGAGATAAGG-3′ and 5’-TTTCATTTGTTCATTTTGTACAACC-3′, PF3D7_1476500 5’-CTTCGATTCACAAATGCAGAAA-3′ and 5’-CGTATTCCACACCTTGTTCTAT-3′, PF3D7_0717700 5’-AAGTAGCAGGTCATCGTGGTT-3′ and 5’-GTTCGGCACATTCTTCCATAA-3′.

## Additional files

Additional file 1: Masking of *P. falciparum* genome annotation file to exclude polymorphic regions of genes. (PDF 546 kb)

Additional file 2: **Table S1.** List of genes with different detected transcript levels between 3D7 and D10 strains by absolute log2 fold > 2 (at least 4 fold difference). Note that this list contains some genes that are deleted in either line. There are also two highly polymorphic genes that were not masked in the initial annotation file, asterisked below (*) and excluded from subsequent analyses. (XLSX 18 kb)

Additional file 3: **Figure S1.** Multiple biological replicate preparations of *P. falciparum* schizont stage transcriptomes in correlation with reference stage-specific transcriptome data. Multiple parasite preparations were made from four long-term laboratory adapted clones (between seven and ten replicates of each) and six Ghanaian clinical isolates (five or six replicates of each) for RNA-seq analysis. Each parasite culture was enriched for schizont-stage parasites, with egress blocked using E64 treatment. Plots show the correlations of FPKM values across all genes in comparisons with previous data from seven time-points across the *P. falciparum* asexual erythrocytic cycle [33], with peak correlations indicating the predominant parasite stage in each replicate. Red lines plot data for samples with peak correlation at either 40 or 48 h post-invasion, and grey lines plot replicate samples that did not maximally correlate with either of these time points, which were therefore excluded from further analysis. (TIFF 92 kb)

Additional file 4: **Table S2.** Genes showing significant differences in transcript levels (log2 fold > 2, at least 4 fold difference on average) in comparison of six cultured clinical isolates and four long-term laboratory adapted clones, including all biological replicate samples. For genes among the top quartile of expression values genome-wide (top 18 genes in the table), members of multigene families and genes in which strain-specific deletions may be responsible for differences are annotated with asterisks (*). (XLSX 20 kb)

Additional file 5: **Figure S2.** Normalised read counts for the ten most highly differentiated genes between cultured clinical isolates and laboratory-adapted clones. Individual sample replicates are plotted according to the time of overall peak transcriptome correlation with reference time course data (either 40 or 48 h). Replicates from clinical isolates are in red (those having peak correlation with 40 h are plotted), and replicates from laboratory isolates are in blue. (PDF 312 kb)

Additional file 6: **Table S3.** Log_2_ fold differences in transcript levels of genes differentially expressed among pairwise comparisons of six cultured clinical isolates with multiple schizont preparations of each, among genes within the top quartile of expression overall. (XLSX 24 kb)

Additional file 7: **Figure S3.** Differential expression of merozoite invasion-related genes among schizonts from different parasite cultures. Distributions of read counts (normalised to library size) for eight genes, for replicated laboratory-adapted and clinical isolate samples, showing data from each replicate culture preparation of each strain. (PDF 324 kb)

Additional file 8: **Figure S3.** Gene expression levels for eight genes newly detected as differentially expressed among clinical isolates (Table 1). Distributions of read counts (normalised to library size) for eight genes, showing data from each replicate culture preparation of each strain. (PDF 84 kb)

Additional file 9: Sequence annotation file Pf3D7.May2015.NoSplice.LSHTM.gtf (see Additional file 1 for explanatory details). (GTF 2322 kb)

Additional file 10: Sequence annotation file GTF_VarRifStev_filtered out.gtf (see Additional file 1 for explanatory details). (GTF 2102 kb)

Additional file 11: RNA-seq comparison of gene expression in paired E64-treated and untreated *P. falciparum* 3D7 schizont preparations. (PDF 365 kb)
