# Supplementary material for: Schizont transcriptome variation among clinical isolates and laboratory-adapted clones of the malaria parasite Plasmodium falciparum
Source: BMC Genomics. 2018 Dec 10;19:894. doi: 10.1186/s12864-018-5257-x (PMC6288915; doi:10.1186/s12864-018-5257-x)
Supplement: Supplementary file 1 — Masking of P. falciparum genome annotation file to exclude polymorphic regions of genes. (PDF 546 kb) [file 12864_2018_5257_MOESM1_ESM.pdf]

Supplementary Materials and Methods

Masking of *P. falciparum* genome annotation file for polymorphic regions of genes

The *P. falciparum* v3.1 genome annotation file (accessed May 2015) was masked for regions of polymorphism. To this end, schizont-stage RNA-seq reads for nine clinical isolates from Ghana (INV018, INV020, INV027, INV032, INV051, INV054, INV055, INV056 and INV060) were viewed in Artemis (Rutherford *et al.* 2000, release version 15.1.4), alongside a schizont stage transcriptome of 3D7 taken at 40 hours post invasion. The following parameters were used when viewing the transcriptome data: proper pair reads were shown, unmapped reads were hidden, a read mapping quality of 5 was applied, the coverage view option was selected and within the 'coverage options' section a manual window size of 1 was used. A polymorphic region was then defined as being a region of 100 base pairs or more where the read depth dropped from at least 50 (including other exons of the same gene) to zero in at least one but not all of the ten parasite samples being analysed. Polymorphic regions were not annotated for *var*, *rifin*, *stevor* or non-coding RNA regions, as these were not included in analysis in the current study. Where multiple isolates fulfilled the above criteria, regions were combined across isolates to span the most distal start and end drop-off points. A gene could have more than one distinct polymorphic region called. Using these parameters, each chromosome was visually scanned and manually scored independently by at least two investigators. The chromosome, gene ID and start and end positions of all prospective regions were recorded. All genes containing potential polymorphic regions were subsequently re-examined by one investigator, to confirm they satisfied the above criteria finalise recorded boundaries to produce a final list of 176 regions defined within 139 different genes across 14 chromosomes (Regions Data List A). These regions were masked from exons in the original *P. falciparum* May 2015 (v3.1) genome annotation file.

This first annotation file (Additional file S9: Pf3D7.May2015.NoSplice.LSHTM.gtf) was further corrected for gene ID and base annotations, and masked for other regions of known polymorphism (Miles *et al.* 2016), genes from multi-gene families known to be highly polymorphic, and duplicated genes. For 500 bp windows, the median nucleotide diversity was calculated using data from three *P. falciparum* crosses (3D7 x HB3, HB3 x Dd2 and 7G8 x

GB4, Miles *et al.* 2016). Windows for which the median nucleotide diversity was greater than 0.025 were classed as polymorphic. Twenty-nine regions (within 24 genes and two intergenic regions; Regions Data List B) were masked from the annotation file by manual annotation.

Var, *rifin* and *stevor* genes were removed from the genome annotation file, since the hypervariable nature of these large, highly polymorphic multi-gene families limit their usefulness in transcriptomic comparisons between isolates for which curated genome sequences are not available. The *Plasmodium* genome database (PlasmoDB v32) was searched for genes containing "var OR rifin OR stevor". The resulting list of 357 genes was manually curated to remove any 'associated' proteins. The remaining 325 genes were removed from the annotation file using the Galaxy "Filter GTF data by attribute values_list" tool (https://usegalaxy.org/). Finally, the annotation file was manually curated to remove duplicated genes. The final annotation file is Additional file S10 (GTF_VarRifStev_filtered out.gtf), which was used to mask the reference genome for subsequent mapping of Illumina short read RNA-seq data.

Regions Data List A

Regions of polymorphism as determined by examination of RNA-seq read drop-off in schizont-stage clinical isolates.

Chromosome	Gene ID	Start Position	End Position	Region Size	CDS Length	Region Proportion of CDS (%)
**1**	PF3D7_0102200	101791	102031	240	3258	7.37
**1**	PF3D7_0104300	190861	190991	130	10500	1.24
**1**	PF3D7_0104300	192558	192678	120	10500	1.14
**1**	PF3D7_0108300	338178	338342	164	6666	2.46
**1**	PF3D7_0108500	349523	349704	181	540	33.52
**1**	PF3D7_0108700	353849	353993	144	3459	4.16
**1**	PF3D7_0110600	406218	406347	129	5133	2.51
**1**	PF3D7_0110600	407033	407150	117	5133	2.28
**1**	PF3D7_0110600	408477	408610	133	5133	2.59
**2**	PF3D7_0201900	91558	91838	280	7326	3.82
**2**	PF3D7_0203000	145207	145327	120	5940	2.02
**2**	PF3D7_0206500	264146	264347	201	4311	4.66
**2**	PF3D7_0206800	273892	274375	483	819	58.97
**2**	PF3D7_0207100	283457	283655	198	5238	3.78
**2**	PF3D7_0207600	306134	306255	121	2994	4.04
**2**	PF3D7_0208400	344111	344253	142	6033	2.35
**2**	PF3D7_0209500	392672	392795	123	1515	8.12
**2**	PF3D7_0212100	484222	484411	189	5535	3.41
**2**	PF3D7_0220000	797917	798601	684	4677	14.62
**3**	PF3D7_0302200	120409	120974	565	4251	13.29
**3**	PF3D7_0302200	122291	123059	768	4251	18.07
**3**	PF3D7_0302200	123267	124497	1230	4251	28.93
**3**	PF3D7_0302500	135415	136276	861	4254	20.24
**3**	PF3D7_0302500	136807	136951	144	4254	3.39
**3**	PF3D7_0302500	138274	138427	153	4254	3.60
**3**	PF3D7_0302500	139173	140608	1435	4254	33.73
**3**	PF3D7_0307700	331025	331130	105	5481	1.92
**3**	PF3D7_0310200	429678	429801	123	13653	0.90
**3**	PF3D7_0310200	430561	430708	147	13653	1.08
**3**	PF3D7_0310200	434885	435112	227	13653	1.66
**3**	PF3D7_0310200	438562	439180	618	13653	4.53
**3**	PF3D7_0310200	439211	439391	180	13653	1.32
**3**	PF3D7_0317300	699981	700267	286	10185	2.81
**3**	PF3D7_0317700	734635	734819	184	8613	2.14
**3**	PF3D7_0321800	913480	913708	228	8121	2.81
**4**	PF3D7_0402200	132240	132775	535	6660	8.03
**4**	PF3D7_0402200	133404	133541	137	6660	2.06
**4**	PF3D7_0404300	235570	235673	103	1896	5.43
**4**	PF3D7_0404600	249585	249752	167	12417	1.34
**4**	PF3D7_0406500	335938	336093	155	9636	1.61

**4**	PF3D7_0406500	340679	340990	311	9636	3.23
**4**	PF3D7_0406500	342457	343064	607	9636	6.30
**4**	PF3D7_0407800	388337	388484	147	5883	2.50
**4**	PF3D7_0410000	466383	466490	107	2502	4.28
**4**	PF3D7_0419900	879351	879642	291	15108	1.93
**4**	PF3D7_0419900	888000	888186	186	15108	1.23
**4**	PF3D7_0420300	924730	924891	161	10422	1.54
**4**	PF3D7_0423600	1064348	1064520	172	4839	3.55
**5**	PF3D7_0501300	69901	70034	133	1014	13.12
**5**	PF3D7_0505100	224845	224946	101	8265	1.22
**5**	PF3D7_0506500	265607	267458	1851	11943	15.50
**5**	PF3D7_0506900	290687	290852	165	2280	7.24
**5**	PF3D7_0507800	325306	325449	143	4584	3.12
**5**	PF3D7_0508100	333352	333467	115	5025	2.29
**5**	PF3D7_0508900	369309	369518	209	9405	2.22
**5**	PF3D7_0508900	374515	374874	359	9405	3.82
**5**	PF3D7_0508900	375083	375199	116	9405	1.23
**5**	PF3D7_0509100	384874	385018	144	5127	2.81
**5**	PF3D7_0510200	437621	437756	135	2244	6.02
**5**	PF3D7_0513600	577399	577515	116	3342	3.47
**5**	PF3D7_0515300	631846	631950	104	6402	1.62
**5**	PF3D7_0516800	705232	705475	243	7137	3.40
**5**	PF3D7_0517400	732758	732996	238	3426	6.95
**5**	PF3D7_0527500	1144946	1145148	202	1377	14.67
**6**	PF3D7_0613800	566707	566895	188	12330	1.52
**6**	PF3D7_0613800	567470	567625	155	12330	1.26
**6**	PF3D7_0613800	569866	569975	109	12330	0.88
**6**	PF3D7_0613800	572601	572763	162	12330	1.31
**6**	PF3D7_0615400	638835	638982	147	8190	1.79
**6**	PF3D7_0624600	1002215	1002352	137	8160	1.68
**6**	PF3D7_0628200	1167365	1167522	157	9219	1.70
**7**	PF3D7_0704300	202029	202271	242	5559	4.35
**7**	PF3D7_0707300	339803	340277	474	2586	18.33
**7**	PF3D7_0710000	451547	451690	143	9804	1.46
**7**	PF3D7_0710200	463817	464018	201	8733	2.30
**7**	PF3D7_0710200	465104	465206	102	8733	1.17
**7**	PF3D7_0710200	465646	466360	714	8733	8.18
**7**	PF3D7_0710200	467869	468672	803	8733	9.20
**7**	PF3D7_0710200	469853	470115	262	8733	3.00
**7**	PF3D7_0710200	470363	470702	339	8733	3.88
**7**	PF3D7_0716000	706083	706286	203	3378	6.01
**7**	PF3D7_0723800	996606	996858	252	6573	3.83
**7**	PF3D7_0724600	1031890	1031992	102	6126	1.67
**7**	PF3D7_0725100	1065916	1066189	273	4731	5.77
**7**	PF3D7_0725100	1067857	1067991	134	4731	2.83
**7**	PF3D7_0727800	1177899	1178099	200	5757	3.47
**7**	PF3D7_0728600	1222709	1222970	261	6489	4.02

**7**	PF3D7_0730900	1329124	1329823	699	6333	11.04
**8**	PF3D7_0803500	218832	219049	217	4404	4.93
**8**	PF3D7_0807100	375154	375295	141	3966	3.56
**8**	PF3D7_0807100	375494	375623	129	3966	3.25
**8**	PF3D7_0807600	392146	392378	232	4005	5.79
**8**	PF3D7_0812100	608784	608892	108	7956	1.36
**8**	PF3D7_0818900	862283	862417	134	2034	6.59
**8**	PF3D7_0829000	1248451	1248597	146	5724	2.55
**8**	PF3D7_0831600	1362696	1362814	118	4185	2.82
**8**	PF3D7_0831800	1374305	1374836	531	918	57.84
**9**	PF3D7_0919900	816122	816242	120	10146	1.18
**9**	PF3D7_0924500	996754	996894	140	3897	3.59
**9**	PF3D7_0930300	1201982	1202175	193	5163	3.74
**9**	PF3D7_0930300	1202644	1202895	251	5163	4.86
**9**	PF3D7_0930300	1204005	1204121	116	5163	2.25
**9**	PF3D7_0930500	1214059	1214207	148	4083	3.62
**9**	PF3D7_0935200	1370958	1371084	126	3444	3.66
**10**	PF3D7_1008100	332821	332928	107	11562	0.93
**10**	PF3D7_1017600	707647	707806	159	2916	5.45
**10**	PF3D7_1019100	771214	771355	141	5787	2.44
**10**	PF3D7_1019700	802554	802659	105	3225	3.26
**10**	PF3D7_1023900	1005560	1005677	117	9987	1.17
**10**	PF3D7_1023900	1005916	1006059	143	9987	1.43
**10**	PF3D7_1033100	1326014	1326123	109	4305	2.53
**10**	PF3D7_1035100	1391759	1391942	183	1686	10.85
**10**	PF3D7_1035200	1395119	1396485	1366	1758	77.70
**10**	PF3D7_1035300	1402123	1402237	114	3702	3.08
**10**	PF3D7_1035400	1404344	1404697	353	1065	33.15
**10**	PF3D7_1035500	1407521	1407785	264	1116	23.66
**10**	PF3D7_1035700	1413654	1414474	820	2094	39.16
**10**	PF3D7_1035900	1424700	1424811	111	1701	6.53
**10**	PF3D7_1035900	1425131	1425381	250	1701	14.70
**11**	PF3D7_1107300	310416	310518	102	10005	1.02
**11**	PF3D7_1117900	681301	681406	105	4416	2.38
**11**	PF3D7_1123000	898128	898231	103	7404	1.39
**11**	PF3D7_1130700	1182465	1182662	197	5457	3.61
**11**	PF3D7_1131800	1228081	1228192	111	4731	2.35
**11**	PF3D7_1132400	1258613	1258740	127	4386	2.90
**11**	PF3D7_1133400	1294318	1294839	521	1869	27.88
**11**	PF3D7_1136000	1407873	1408113	240	10407	2.31
**11**	PF3D7_1149000	1953727	1953833	106	18282	0.58
**11**	PF3D7_1149000	1955409	1955648	239	18282	1.31
**11**	PF3D7_1149000	1958705	1958972	267	18282	1.46
**11**	PF3D7_1149000	1963139	1963294	155	18282	0.85
**11**	PF3D7_1149200	1977853	1977992	139	3273	4.25
**12**	PF3D7_1209300	430302	430564	262	4386	5.97
**12**	PF3D7_1211800	526944	527171	227	1146	19.81

**12**	PF3D7_1202600	149525	149720	195	6543	2.98
**12**	PF3D7_1227200	1098835	1098948	113	5901	1.91
**12**	PF3D7_1227500	1120159	1120260	101	6912	1.46
**12**	PF3D7_1228600	1160721	1160918	197	2232	8.83
**12**	PF3D7_1228800	1176357	1176819	462	9630	4.80
**12**	PF3D7_1229600	1216859	1217213	354	3492	10.14
**12**	PF3D7_1235900	1499821	1499984	163	3096	5.26
**12**	PF3D7_1238500	1598381	1598516	135	5439	2.48
**12**	PF3D7_1248700	1996055	1996159	104	5970	1.74
**13**	PF3D7_1329600	1259388	1259501	113	906	12.47
**13**	PF3D7_1335100	1419294	1419454	160	1056	15.15
**13**	PF3D7_1335300	1431202	1438587	7385	9765	75.63
**13**	PF3D7_1335400	1441061	1448396	7335	9393	78.09
**13**	PF3D7_1337500	1515603	1515875	272	10005	2.72
**13**	PF3D7_1339700	1593564	1593835	271	5811	4.66
**13**	PF3D7_1348400	1945641	1946256	615	11568	5.32
**13**	PF3D7_1356800	2260273	2260378	105	12135	0.87
**13**	PF3D7_1357000	2266094	2266806	712	1332	53.45
**13**	PF3D7_1357100	2269140	2269737	597	1332	44.82
**13**	PF3D7_1362700	2513983	2514092	109	6120	1.78
**13**	PF3D7_1362700	2514345	2514619	274	6120	4.48
**13**	PF3D7_1366400	2658121	2658265	144	3789	3.80
**13**	PF3D7_1367700	2696594	2696715	121	4227	2.86
**14**	PF3D7_1402800	102123	102294	171	4536	3.77
**14**	PF3D7_1403100	114151	114726	575	5868	9.80
**14**	PF3D7_1410300	412342	412456	114	13218	0.86
**14**	PF3D7_1411000	440789	440985	196	6165	3.18
**14**	PF3D7_1412400	496454	496924	470	9657	4.87
**14**	PF3D7_1413700	542183	542324	141	3642	3.87
**14**	PF3D7_1423800	969884	970052	168	4407	3.81
**14**	PF3D7_1425400	990402	990591	189	3711	5.09
**14**	PF3D7_1428900	1136859	1136979	120	4338	2.77
**14**	PF3D7_1431300	1231562	1231853	291	2502	11.63
**14**	PF3D7_1439500	1609844	1609971	127	3225	3.94
**14**	PF3D7_1442900	1755336	1755542	206	10155	2.03
**14**	PF3D7_1448500	1989515	1989837	322	11115	2.90
**14**	PF3D7_1452000	2135670	2135792	122	6570	1.86
**14**	PF3D7_1452400	2149153	2149356	203	2703	7.51
**14**	PF3D7_1464500	2613108	2613233	125	9756	1.28
**14**	PF3D7_1468100	2791803	2791923	120	7677	1.56
**14**	PF3D7_1472200	2947686	2947858	172	6756	2.55
**14**	PF3D7_1472200	2948840	2949028	188	6756	2.78

Regions Data List B

Regions of high nucleotide diversity (> 0.025) based on Miles *et al.* 2016

Gene ID	Chromosome	Window start	Window end
**PF3D7_0104100**	Pf3D7_01_v3	180001	180500
**PF3D7_0113600**	Pf3D7_01_v3	513001	513500
**PF3D7_0113600**	Pf3D7_01_v3	514001	515000
**PF3D7_0113800**	Pf3D7_01_v3	530001	531500
**PF3D7_0207600**	Pf3D7_02_v3	306001	306500
**PF3D7_0221000**	Pf3D7_02_v3	848501	849000
**PF3D7_0302300**	Pf3D7_03_v3	129501	130500
**PF3D7_0401600.2**	Pf3D7_04_v3	92501	93000
**PF3D7_0402200**	Pf3D7_04_v3	128001	128500
**PF3D7_0402200**	Pf3D7_04_v3	132001	134000
**PF3D7_0424400**	Pf3D7_04_v3	1101001	1102000
**PF3D7_0425200**	Pf3D7_04_v3	1137001	1138000
**PF3D7_0505000**	Pf3D7_05_v3	216501	217000
**intergenic**	Pf3D7_07_v3	78001	78500
**PF3D7_0701900**	Pf3D7_07_v3	79501	80500
**PF3D7_0710200**	Pf3D7_07_v3	466001	466500
**PF3D7_0713100**	Pf3D7_07_v3	606001	606500
**PF3D7_0830800**	Pf3D7_08_v3	1311001	1312500
**PF3D7_0831600**	Pf3D7_08_v3	1362501	1363000
**PF3D7_0930300**	Pf3D7_09_v3	1202001	1202500
**PF3D7_0930300**	Pf3D7_09_v3	1203001	1207000
**PF3D7_1035200**	Pf3D7_10_v3	1394501	1395000
**intergenic**	Pf3D7_10_v3	1397001	1398500
**PF3D7_1035700**	Pf3D7_10_v3	1413501	1414500
**PF3D7_1036300**	Pf3D7_10_v3	1433001	1434500
**PF3D7_1301800**	Pf3D7_13_v3	106001	107000
**PF3D7_1422900**	Pf3D7_14_v3	922501	923000
**PF3D7_1475800**	Pf3D7_14_v3	3121001	3121500
**PF3D7_1477600**	Pf3D7_14_v3	3193501	3194500

References

Kim D, Langmead B and Salzberg SL. HISAT: a fast spliced aligner with low memory requirements. *Nature Methods* 2015

Li H, Handsaker B, Wysoker A, Fennell T, Ruan J, Homer N, Marth G, Abecasis G, Durbin R and 1000 Genome Project Data Processing Subgroup. The Sequence alignment/map (SAM) format and SAMtools. *Bioinformatics* 2009

Rutherford K, Parkhill J, Crook J, Horsnell T, Rice P, Rajandream MA and Barrell B. Artemis: sequence visualization and annotation. *Bioinformatics* 2000

Broadbent KM, Broadbent JC, Ribacke U, Wirth D, Rinn JL and Sabeti PC. Strand-specific RNA sequencing in *Plasmodium falciparum* malaria identifies developmentally regulated long non-coding RNA and circular RNA. *BMC Genomics* 2015

Lawrence M, Huber W, Pagès H, Aboyoun P, Carlson M, Gentleman R, Morgan M and Carey V. Software for Computing and Annotating Genomic Ranges. *PLoS Computational Biology,* 2013

Love MI, Huber W and Anders S. Moderated estimation of fold change and dispersion for RNA-seq data with DESeq2. *Genome Biology* 2014

Rutherford K, Parkhill J, Crook J, Horsnell T, Rice P, Rajandream MA and Barrell B. Artemis: sequence visualization and annotation. *Bioinformatics* 2000

Miles A, Iqbal Z,Vauterin P, Pearson R, Campino S, Theron M, Gould K, Mead D, Drury E, O'Brien J, Ruano Rubio V, MacInnis B, Mwangi J, Samarakoon U, Ranford-Cartwright L, Ferdig M, Hayton K, Su X-Z Wellems T, Rayner J, McVean G, Kwiatkowski D. Indels, structural variation, and recombination drive genomic diversity in *Plasmodium falciparum*. *Genome Research* 2016
