# Supplementary figures and images for: Schizont transcriptome variation among clinical isolates and laboratory-adapted clones of the malaria parasite Plasmodium falciparum
Source: BMC Genomics. 2018 Dec 10;19:894. doi: 10.1186/s12864-018-5257-x (PMC6288915; doi:10.1186/s12864-018-5257-x)

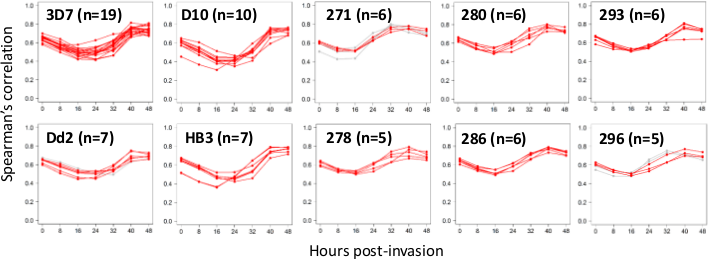

Supplement: Supplementary file 3 — Figure S1. Multiple biological replicate preparations of P. falciparum schizont stage transcriptomes in correlation with reference stage-specific transcriptome data. Multiple parasite preparations were made from four long-term laboratory adapted clones (between seven and ten replicates of each) and six Ghanaian clinical isolates (five or six replicates of each) for RNA-seq analysis. Each parasite culture was enriched for schizont-stage parasites, with egress blocked using E64 treatment. Plots show the correlations of FPKM values across all genes in comparisons with previous data from seven time-points across the P. falciparum asexual erythrocytic cycle [33], with peak correlations indicating the predominant parasite stage in each replicate. Red lines plot data for samples with peak correlation at either 40 or 48 h post-invasion, and grey lines plot replicate samples that did not maximally correlate with either of these time points, which were therefore excluded from further analysis. (TIFF 92 kb) [file 12864_2018_5257_MOESM3_ESM.tiff]

Normalised counts

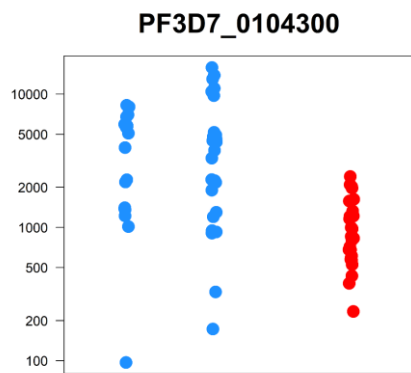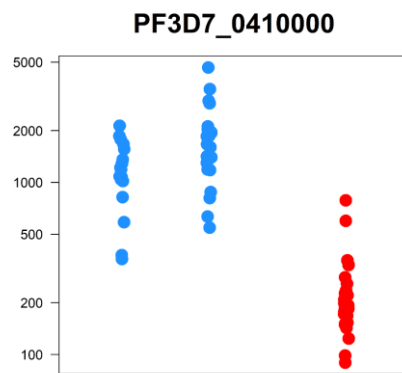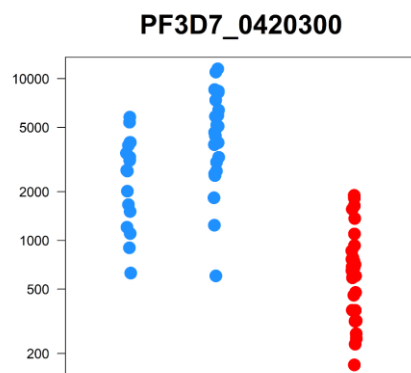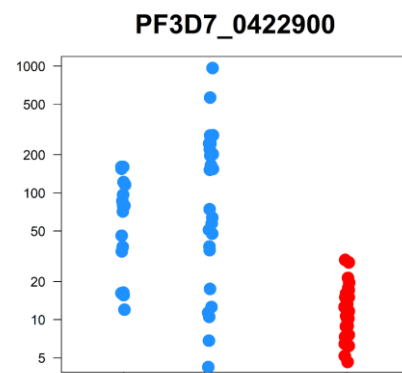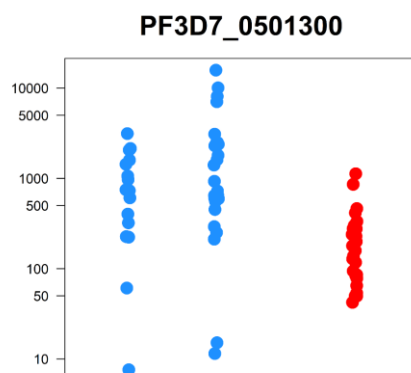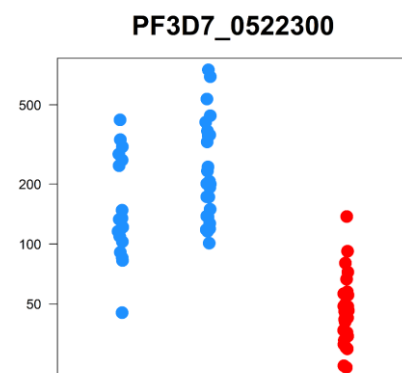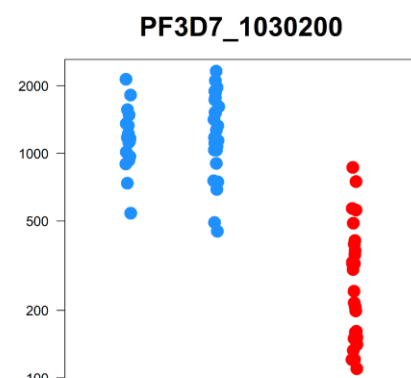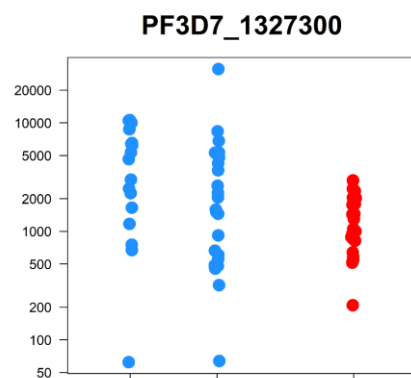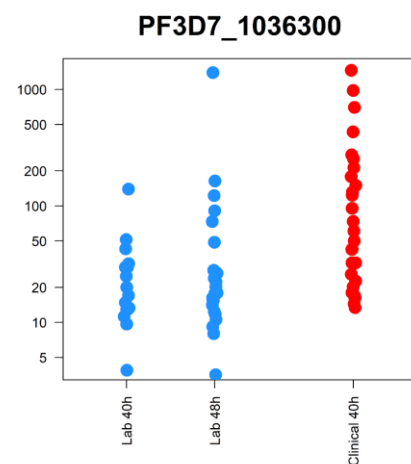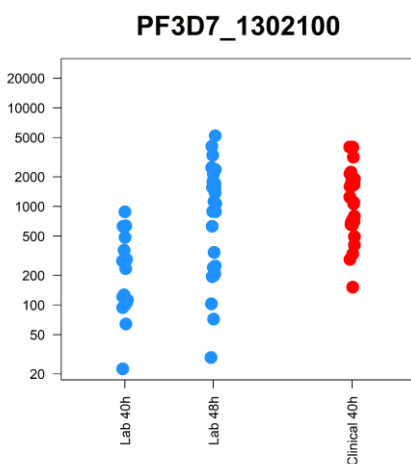

Supplement: Supplementary file 5 — Figure S2. Normalised read counts for the ten most highly differentiated genes between cultured clinical isolates and laboratory-adapted clones. Individual sample replicates are plotted according to the time of overall peak transcriptome correlation with reference time course data (either 40 or 48 h). Replicates from clinical isolates are in red (those having peak correlation with 40 h are plotted), and replicates from laboratory isolates are in blue. (PDF 312 kb) [file 12864_2018_5257_MOESM5_ESM.pdf]

**EBA-140**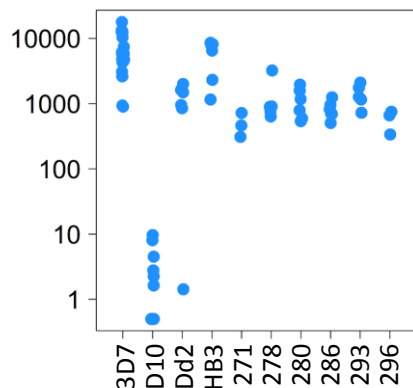**EBA-175**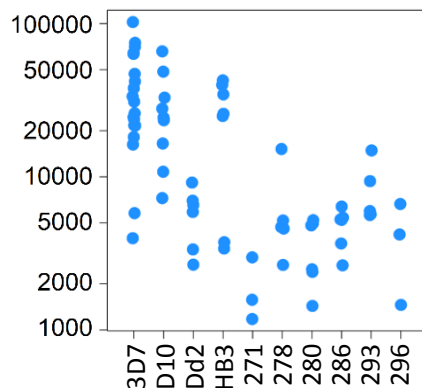**EBA-181**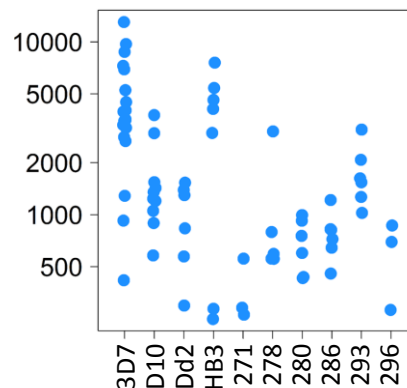**RH1**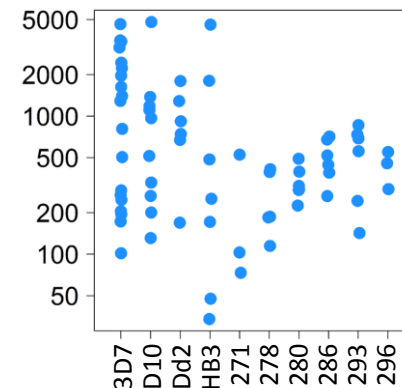**RH2a**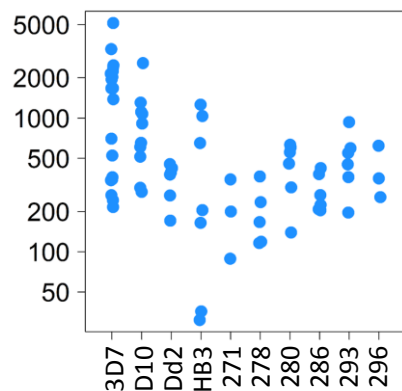**RH2b**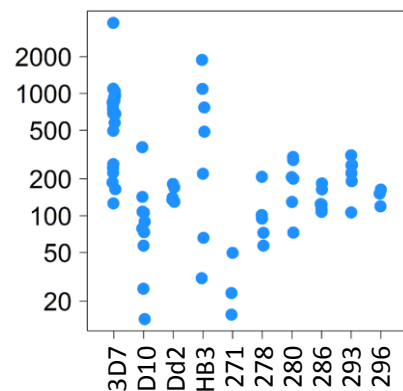**RH4**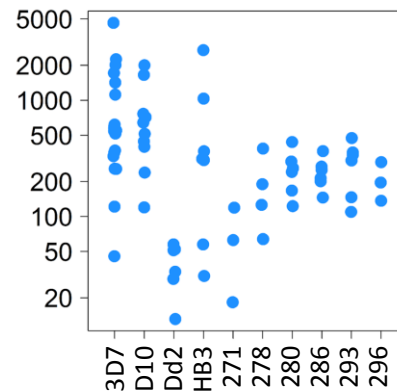**RH5**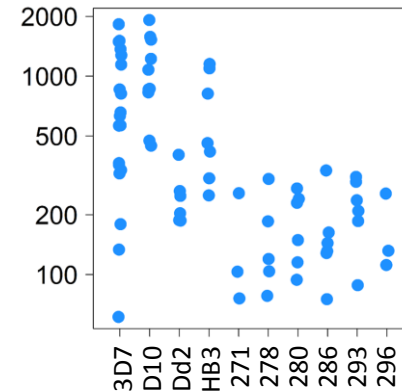

Supplement: Supplementary file 7 — Figure S3. Differential expression of merozoite invasion-related genes among schizonts from different parasite cultures. Distributions of read counts (normalised to library size) for eight genes, for replicated laboratory-adapted and clinical isolate samples, showing data from each replicate culture preparation of each strain. (PDF 324 kb) [file 12864_2018_5257_MOESM7_ESM.pdf]

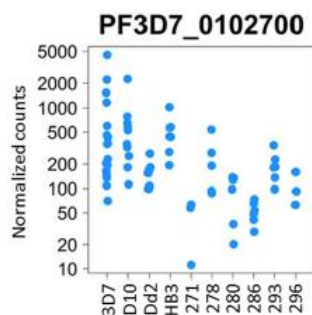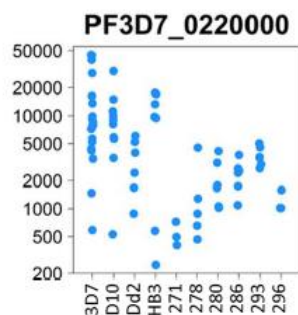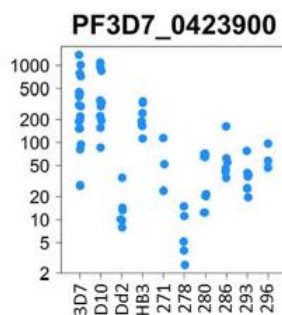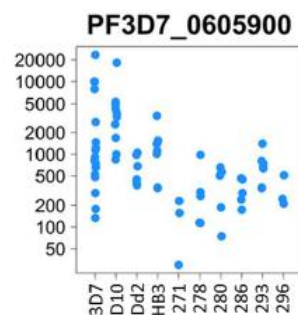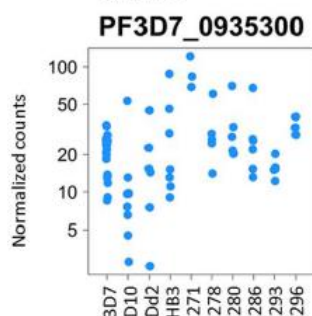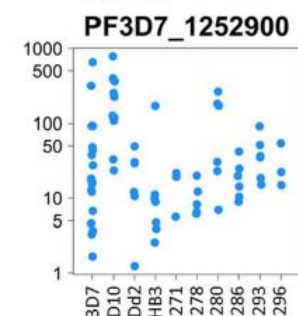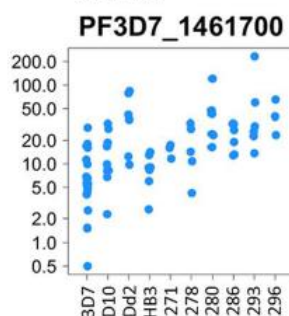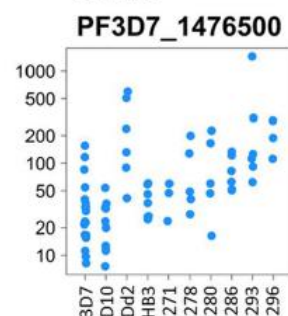

Supplement: Supplementary file 8 — Figure S3. Gene expression levels for eight genes newly detected as differentially expressed among clinical isolates (Table 1). Distributions of read counts (normalised to library size) for eight genes, showing data from each replicate culture preparation of each strain. (PDF 84 kb) [file 12864_2018_5257_MOESM8_ESM.pdf]
