# Supplementary material for: Schizont transcriptome variation among clinical isolates and laboratory-adapted clones of the malaria parasite Plasmodium falciparum
Source: BMC Genomics. 2018 Dec 10;19:894. doi: 10.1186/s12864-018-5257-x (PMC6288915; doi:10.1186/s12864-018-5257-x)
Supplement: Supplementary file 11 — RNA-seq comparison of gene expression in paired E64-treated and untreated P. falciparum 3D7 schizont preparations. (PDF 365 kb) [file 12864_2018_5257_MOESM11_ESM.pdf]

Analysis of paired E64-treated and untreated *P. falciparum* 3D7 schizonts

Methods

In order to compare the effect of E64 on parasite transcriptomes, four separate replicates of schizont-stage parasites were incubated with 10 µM E64 (or PBS control) for 5.5 hours in the presence of uninfected erythrocytes, and compared with four separate replicate controls that did not employ E64. Schizonts were purified as described in Materials and Methods, with the exception that untreated cultures were prepared using three times the volume of culture compared with E64-treated cultures, to ensure that untreated cultures contained sufficient remaining schizont-stage material for RNA-seq, following parasite egress over the course of the treatment. Following the treatment, schizonts were percoll purified and RNA was extracted and sequenced in accordance with main materials and methods.

Analysis summary

Log$_2$ FPKM values for samples and replicates were all strongly positively correlated by both Spearman and Pearson correlations (Correlations Summary 1) with significant p-values (Correlations Summary 2). Differential gene expression analysis was conducted using DEseq2 with the design formula design = ~ Treatment + Replicate.

Correlations Summary 1
Spearman and Pearson correlations of E64-treated and untreated replicate.

	Rep1-E64	Rep1-Untreated	Rep2-E64	Rep2-Untreated	Rep3-E64	Rep3-Untreated	Rep4-E64	Rep4-Untreated	
**Rep1-E64**		1	0.952381	0.97619	0.952381	0.928571	0.904762	0.97619	**Spearman**
**Rep1-Untreated**	0.997363		0.952381	0.97619	0.952381	0.928571	0.904762	0.97619	
**Rep2-E64**	0.824789	0.811063		0.97619	0.952381	0.857143	0.880952	0.928571	
**Rep2-Untreated**	0.900262	0.891954	0.979414		0.97619	0.904762	0.928571	0.952381	
**Rep3-E64**	0.991939	0.989546	0.86838	0.932429		0.952381	0.97619	0.97619	
**Rep3-Untreated**	0.990177	0.995798	0.784867	0.875363	0.984077		0.97619	0.97619	
**Rep4-E64**	0.911197	0.903819	0.908583	0.942909	0.950347	0.908216		0.952381	
**Rep4-Untreated**	0.912057	0.910578	0.940235	0.974183	0.947738	0.911399	0.983432		
	**Pearson**								

Correlations Summary 2
Spearman and Pearson p-values for correlations in Table 1.

	Rep1-E64	Rep1-Untreated	Rep2-E64	Rep2-Untreated	Rep3-E64	Rep3-Untreated	Rep4-E64	Rep4-Untreated	
**Rep1-E64**		0.00E+00	2.60E-04	3.31E-05	0.00E+00	6.79E-04	8.63E-04	0.00E+00	**Spearman**
**Rep1-Untreated**	1.62E-07		2.60E-04	3.31E-05	0.00E+00	6.79E-04	8.63E-04	0.00E+00	
**Rep2-E64**	2.49E-04	5.41E-04		3.31E-05	2.60E-04	1.38E-02	1.49E-02	2.60E-04	
**Rep2-Untreated**	2.95E-05	6.41E-05	3.83E-06		3.31E-05	7.47E-03	6.53E-03	3.31E-05	
**Rep3-E64**	9.21E-07	4.15E-06	1.12E-04	2.09E-05		6.79E-04	8.63E-04	0.00E+00	
**Rep3-Untreated**	6.50E-06	5.82E-07	1.17E-03	1.70E-04	1.24E-05		6.55E-05	6.79E-04	
**Rep4-E64**	3.33E-05	6.53E-05	5.42E-04	1.71E-04	1.98E-06	2.89E-05		0.00E+00	
**Rep4-Untreated**	1.55E-05	1.46E-05	1.43E-04	1.42E-05	2.85E-06	5.18E-06	6.06E-06		
	**Pearson**								

Only a single gene (PF3D7_0220700, a sub-telomeric gene encoding an exported protein of unknown function from the *hyp9* family) showed a fold difference greater than log$_2$ of 2.
